# Study of the Structure and Bioactivity of Polysaccharides from Different Parts of *Stemona tuberosa* Lour

**DOI:** 10.3390/molecules29061347

**Published:** 2024-03-18

**Authors:** Xiang Qiu, Yanghui Ou, Shengjia Lu, Yibin Liang, Yali Zhang, Mengjie Li, Gang Li, Hongwei Ma, Yanting Wu, Zhaoyu He, Zhensheng Zhang, Hongliang Yao, Wen-Hua Chen

**Affiliations:** 1 School of Pharmacy and Food Engineering, Wuyi University, Jiangmen 529020, China; qxiang5202023@163.com; 2 Guangdong Key Laboratory of Animal Conservation and Resource Utilization, Institute of Zoology, Guangdong Academy of Sciences, Guangzhou 510260, China; ouyh0807@gmail.com (Y.O.); 15123186435@163.com (S.L.); 13318113021@163.com (Y.L.); zhangyl@giz.gd.cn (Y.Z.); lmj100800@163.com (M.L.); ligang00890089@163.com (G.L.); 13437871411@sina.cn (Y.W.); 13288832466@163.com (Z.H.); zhang15975510562@163.com (Z.Z.); 3 Guangdong Huakangyuan Medicinal Materials Resources Development Co., Ltd., Qingyuan 511500, China; 18818873320@139.com

**Keywords:** *Stemona tuberosa* Lour, different extraction methods, polysaccharides, physicochemical and structural properties, biological activities

## Abstract

The polysaccharides from *Stemona tuberosa* Lour, a kind of plant used in Chinese herbal medicine, have various pharmacological activities, such as anti-inflammatory and antioxidant properties. However, the effects of the extraction methods and the activity of polysaccharides from different parts are still unknown. Therefore, this study aimed to evaluate the effects of different extraction methods on the yields, chemical compositions, and bioactivity of polysaccharides extracted from different parts of *Stemona tuberosa* Lour. Six polysaccharides were extracted from the leaves, roots, and stems of *Stemona tuberosa* Lour through the use of hot water (i.e., SPS-L1, SPS-R1, and SPS-S1) and an ultrasound-assisted method (i.e., SPS-L2, SPS-R2, and SPS-S2). The results showed that the physicochemical properties, structural properties, and biological activity of the polysaccharides varied with the extraction methods and parts. SPS-R1 and SPS-R2 had higher extraction yields and total sugar contents than those of the other SPSs (SPS-L1, SPS-L2, SPS-S1, and SPS-S2). SPS-L1 had favorable antioxidant activity and the ability to downregulate MUC5AC expression. An investigation of the anti-inflammatory properties showed that SPS-R1 and SPS-R2 had greater anti-inflammatory activities, while SPS-R2 demonstrated the strongest anti-inflammatory potential. The results of this study indicated that SPS-L1 and SPS-L2, which were extracted from non-medicinal parts, may serve as potent natural antioxidants, but further study is necessary to explore their potential applications in the treatment of diseases. The positive anti-inflammatory effects of SPS-R1 and SPS-R2 in the roots may be further exploited in drugs for the treatment of inflammation.

## 1. Introduction

*Stemona tuberosa* Lour is a medicinal plant found in the southern area of the Yangtze River Basin in China. It has been used to treat eczema, scabies, pertussis, and other illnesses [1]. In East and Southeast Asia, extracts from *Stemona tuberosa* Lour are employed as household insecticides and as a treatment for respiratory ailments [2]. The roots of *Stemona tuberosa* Lour possess medicinal properties, which include lung moisturization and cough relief. Additionally, they exhibit activity against insects and lice. *Stemona tuberosa* Lour’s roots have a variety of chemical components, including polysaccharides, lignans, sterols, and alkaloids. To date, most studies have focused on an alkaloid extract from *Stemona tuberosa* Lour’s roots [3]. One of its alkaloids (protostemonine) reduces inflammatory cell infiltration and alleviates LPS-induced acute lung injury [4]. The chemical composition of the non-medicinal parts of the leaves and stems is rarely reported.

A polysaccharide is a naturally occurring, safe, and non-toxic macromolecule with strong biological activity [5]. Polysaccharides exhibit antioxidant, anti-inflammatory, antiviral, antibacterial, hypoglycemic, and liver-protective activity [6,7]. The physical and chemical properties of a polysaccharide, including its average molecular weight (*M_w_*), monosaccharide composition, glycosidic bonds, and configuration, significantly influence its activity [8]. It is known that the leaves, flowers, stems, fruits, and roots of plants are rich in polysaccharides [9]. Most studies have concentrated on the edible or medicinal parts of plants, overlooking the medicinal potential of other parts, eventually leading to wastage.

The hot water extraction method is a commonly used extraction method with a low extraction cost and convenient use. The conditions of hot water extraction are generally the following: the ratio of solid to liquid is 1:5–1:30 g/mL, the extraction temperature is 60-100 °C, and the extraction time is 1–4 h [10,11,12]. However, other extraction methods, such as enzyme-, ultrasound-, and microwave-assisted extraction, are also becoming more popular [13,14,15]. Different extraction methods result in changes in the physicochemical attributes and structure of polysaccharides—mainly in their yield, total sugar content, uronic acid content, *M_w_*, monosaccharide composition, surface topography, and thermal stability [16]. Ultrasound-assisted extraction uses the cavitation effect caused by ultrasound to destroy the plant cell wall and promote the rapid entry of solvent into the cell interior to achieve rapid and efficient extraction [17]. Ultrasonic degradation is a good physical method for the production of polysaccharides with a low value of *M_w_*, in which a break in the glycoside bond of the main chain of the polysaccharide occurs [18,19]. Guo et al. obtained the highest polysaccharide extraction yield (10.29%) at a solid–liquid ratio of 1:60 g/mL, an extraction time of 180 min, an extraction temperature of 80 °C, and an ultrasonic power of 144 W through process optimization [20].

The structures of polysaccharides are frequently related to their antioxidant properties [21]. Oxidation is the process of producing reactive oxygen species (ROSs) in living organisms, including the free radicals 2,2-diphenyl-1-picrylhydrazyl (DPPH), hydroxyl, and 2,2′-azino-bis(3-ethylbenzothiazoline-6-sulfonic acid) (ABTS) [22]. Oxidative stress caused by excessive ROSs can cause damage to tissues and organs, and excessive accumulation of ROSs in organisms can also lead to aging [23]. The scavenging of free radicals is associated with the alleviation of the impact of oxidative stress on cells [24].

Macrophages, as the key effector cells of the innate immune system, have the functions of phagocytosis, secretion, and antigen presentation to protect the host from infection and invasion by external pathogens [25]. Lipopolysaccharides (LPSs) can induce the production of various pro-inflammatory mediators and factors, such as NO, IL-6, IL-1β, and tumor necrosis factor-α (TNF-α). As an important inflammatory mediator, NO is also a very potent free radical that plays an important role in the immune system [26,27]. Detecting the production of NO, IL-6, IL-1β, and TNF-α when stimulated by LPSs is a primary method used to study the anti-inflammatory effects of polysaccharides [28].

The primary mucin produced by cup cells is called MUC5AC, and it is strongly linked to the development of numerous respiratory conditions, including bronchiectasis, asthma, and pulmonary cystic fibrosis [29]. The epidermal growth factor (EGF)-induced NCI-H292 cell (a human lung mucoepidermoid cell line) model is frequently utilized in research with this mucin [30,31,32].

There are few studies on the separation and activity of polysaccharides from the medicinal parts of *Stemona tuberosa* Lour’s roots and even fewer studies on the structural characterization and activity of polysaccharides extracted from the medicinal and non-medicinal parts of *Stemona tuberosa* Lour’s roots, stems, and leaves with different methods. Thus, in this study, we extracted polysaccharides from the stems, leaves, and roots using hot water extraction and ultrasound-assisted extraction, and we compared the physicochemical properties, preliminary structural characterization, anti-oxidative capacity, anti-inflammatory capacity, and MUC5AC expression capacity of these polysaccharides obtained with the two extraction methods; polysaccharides from different parts were also compared. The activity of polysaccharides in different parts may be adapted to different diseases, which provides a feasible example for further purification, structural characterization, in vivo animal experiments, and mechanism studies.

## 2. Results and Discussion

### 2.1. Extraction Yields and Chemical Composition of the SPSs

SPS-L1, SPS-L2, SPS-R1, SPS-R2, SPS-S1, and SPS-S2 represent the polysaccharides that were extracted from the leaves, roots, and stems using hot water extraction and ultrasound-assisted extraction, respectively. Table 1 displays the physicochemical characteristics and extraction yields of the polysaccharides extracted using the different methods as well as from different parts of *Stemona tuberosa* Lour. The extraction yields of the six different types of SPSs were as follows: SPS-R1 (11.75%) > SPS-R2 (11.46%) > SPS-L1 (10.49%) > SPS-L2 (8.75%) > SPS-S1 (7.18%) > SPS-S2 (4.96%). There were significant (*p* < 0.05) differences between SPS-R1, SPS-R2, and the other SPSs.

In comparison with extraction with ultrasonic assistance, the hot water extraction method resulted in higher yields. This difference could be attributed to ultrasound breaking down the polysaccharide chain, leading to a decreased extraction yield [33,34]. Wang et al. reported a higher extraction yield of pectin polysaccharide from *Choerospondias axillaris* peels using hot water extraction than when using ultrasound-assisted extraction [35].

The total sugar content was found to be comparatively high in SPS-R1 and SPS-R2 (89.98% and 80.50%), followed by SPS-S1 (42.23%) and SPS-S2 (41.18%), while SPS-L1 (34.06%) and SPS-L2 (23.30%) showed the lowest total sugar content. This pattern was consistent with the results of the extraction yield, indicating that the total sugar content obtained through hot water extraction surpassed that obtained through ultrasound-assisted extraction [36,37]. It is noted that the polysaccharides from the different parts of *Stemona tuberosa* Lour exhibited significant (*p* < 0.05) variations in total sugar content. The polysaccharides from the roots showed a higher total sugar content than that of the other SPSs. This observation was consistent with the results of the polysaccharide extraction yield, suggesting that, as a medicinal part, the roots contained substantial amounts of polysaccharides. However, the total sugar content of polysaccharides was the lowest in the leaves, which might have been due to the high content of water-soluble impurities and pigments in the leaves [38]. The disparity in total sugar content laid the groundwork for the subsequent analysis of the SPSs’ activities.

The protein content obtained through ultrasound-assisted extraction (SPS-L2 and SPS-S2) was higher than that obtained through hot water extraction (SPS-L1 and SPS-S1); a similar phenomenon was previously documented [37]. The ultrasound process might have promoted the dissolution of proteins. All six types of SPSs were identified as acidic polysaccharides. SPS-L1 and SPS-S1 had higher uronic acid content than that of SPS-L2 and SPS-S2, and similar studies have reported this finding before [39]. In contrast, the opposite tendency was observed in SPS-R1 and SPS-R2. The results showed that hot water extraction and ultrasound-assisted extraction had different effects on the chemical properties of the polysaccharides from different parts. The content of uronic acid in SPS-S1 and SPS-S2 was significantly (*p* < 0.05) higher than that in the other SPSs. This showed that the polysaccharides from the stems were mostly acidic polysaccharides.

### 2.2. Structural Characterization

#### 2.2.1. Molecular Weights of the SPSs

**Figure 1:**
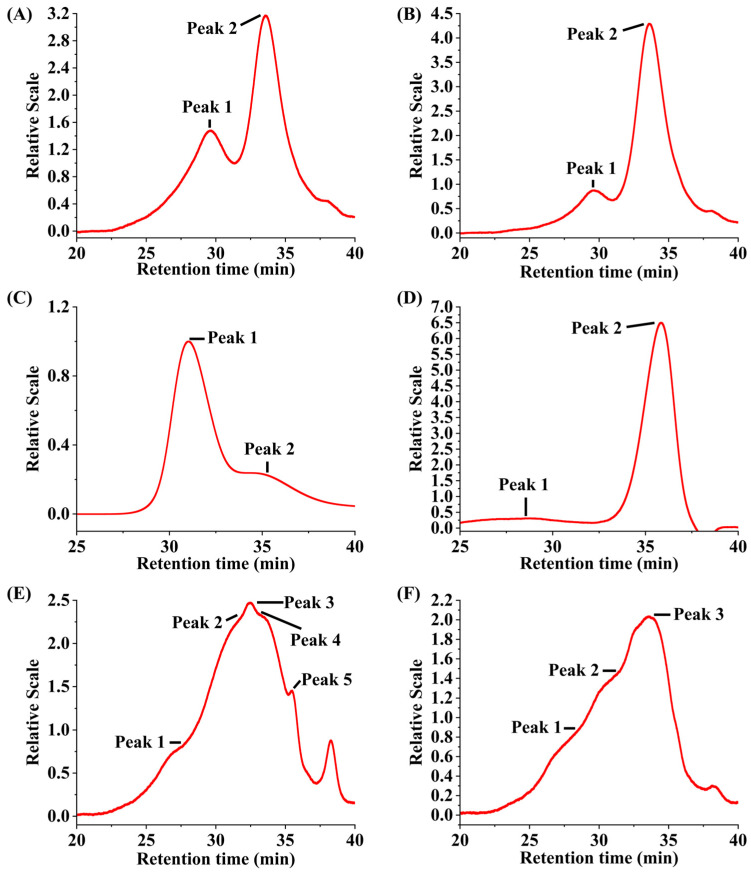
High-performance gel permeation chromatography (HPGPC) of (**A**) SPS-L1, (**B**) SPS-L2, (**C**) SPS-R1, (**D**) SPS-R2, (**E**) SPS-S1, and (**F**) SPS-R2.

Table 2 and Figure 1 show the *M_w_* distribution of the six SPSs. SPS-L1 and SPS-L2 exhibited relatively similar *M_w_* distributions, with low-molecular-weight components of 11,098 Da (63.02%) and 10,834 Da (82.05%) dominating, respectively, and the proportion of components with lower values of *M_w_* increasing. In SPS-R1 and SPS-R2, the components with low values of *M_w_* were predominant. SPS-S1 exhibited components with five different values of *M_w_*: 248,136 Da (11.09%), 27,437 Da (37.65%), 18,918 Da (17.55%), 13,908 Da (24.19%), and 3792 Da (9.52%). For SPS-S2, peak 1 represented a high value of *M_w_* (148,327 Da, 17.18%), peak 2 represented a middle value of *M_w_* (34,035 Da, 24.50%), and peak 3 represented a low value of *M_w_* (10,669 Da, 58.32%). According to the aforementioned results, the *M_w_* values of the dominant components of the SPSs extracted from hot water were higher than those that underwent ultrasound-assisted extraction. This might have been caused by the aggregation of polysaccharides in a hot water environment [40,41]. On the other hand, ultrasonic extraction reduced the *M_w_* values of the polysaccharides by breaking and depolymerizing polymer molecular chains and changing the functional groups of polysaccharides through mechanical physics [42,43]. The *M_w_* values of the dominant components of SPS-R1 and SPS-R2 were lower than those of the other SPSs.

#### 2.2.2. Monosaccharide Composition

The monosaccharide compositions of the six SPSs are shown in Table 3. SPS-L1 and SPS-L2 contained the same types of monosaccharides and had higher amounts of Gal, Man, and GalA, but their amounts were different. The highest percentages of Glc were found to be 69.86% in SPS-R1 and 55.71% in SPS-R2. Table 3 shows the results for the monosaccharide composition; the GalA content of SPS-R2 was higher than that of SPS-R1. However, the types of monosaccharides changed. The predominant monosaccharides in SPS-S1 and SPS-S2 were Gal (20.61%, 22.30%) and GalA (48.82%, 44.62%), with a considerably higher GalA content in these two groups than in SPS-L1, SPS-L2, SPS-R1, and SPS-R2. When compared with the other SPSs, the content of uronic acid in SPS-S1 and SPS-S2 was significantly higher (*p* < 0.05, Table 1). SPS-L1 and SPS-S1 had a higher content of GalA than that of SPS-L2 and SPS-S2, which was consistent with the results shown in Table 1. It has been reported that different extraction processes affect the types of monosaccharides present in polysaccharides and the contents of the monosaccharides [44]. The composition of monosaccharides might be impacted by ultrasonic waves because they cause the hydrolysis of polysaccharide chains and break intermolecular hydrogen bonds [45].

#### 2.2.3. UV Spectroscopic Analysis of the SPSs

**Figure 2:**
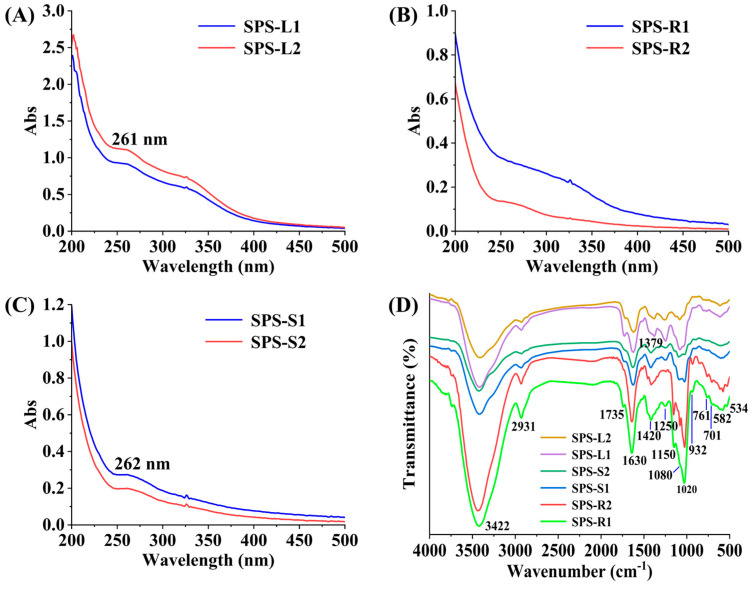
The UV (**A**–**C**) and FT-IR (**D**) spectra of the six SPSs.

Figure 2A–C display the UV spectra of the SPSs. The absorption peaks at 260 nm in SPS-L1, SPS-L2, SPS-S1, and SPS-S2 suggested the potential presence of proteins and nucleic acids in these polysaccharides [37]. SPS-R1 and SPS-R2 had smooth UV spectra, which suggested the presence of very few or no proteins and nucleic acids. The outcome matched the results of the protein detection.

#### 2.2.4. FT-IR Spectra of the SPSs

The FT-IR spectra of the six SPSs are shown in Figure 2D. A strong and wide absorption band at ~3422 cm^−1^ arose from the stretching vibration of O–H. The peaks at ~2931 cm^−1^ represented the stretching vibration of C–H. The absorption peak at ~1735 cm^−1^ was attributed to the stretching vibration of C=O in the esterified carbonyl group (COOR) [46]. The peaks at ~1630 cm^−1^ represented the carbonyl C=O stretching vibrations of terminal sugar residues or uronic acid [47]. This result indicated the presence of uronic acid in the SPSs, which was consistent with the results listed in Table 1. The absorption peak at ~1420 cm^−1^ might have been due to the vibration of amide, and the absorption of SPS-S1 and SPS-S2 at this peak was stronger than that of the other SPSs; the protein content of SPS-S1 and SPS-S2 was also relatively high, as shown in Table 1 [48]. More proteins might also have been present, as the absorption peaks of SPS-L1 and SPS-L2 at 1250 cm^−1^ were stronger than those of the other SPSs, which can also be seen in Table 1 [49]. The absorption peak at 1379 cm^−1^ in SPS-L1 and SPS-L2 might have been due to C-H bending vibrations [50]. The absorption bands at 1020 cm^−1^, ~1080 cm^−1^, and ~1150 cm^−1^ represented the tensile vibration of C–O–C or C–O–H in the SPSs [22]. Moreover, there was a pyramid structure in SPS-R1 and SPS-R2 [51]. The peaks at ~936, 761, and 824 cm^−1^ indicated the presence of β- and α- type glycosidic bonds in the SPSs [52]. The absorption peak at 616 cm^−1^ was attributed to the out-of-plane O-H vibration [53]. In summary, these SPSs exhibited typical polysaccharide absorption peaks in their FT-IR spectra.

#### 2.2.5. SEM Analysis of the SPSs

**Figure 3:**
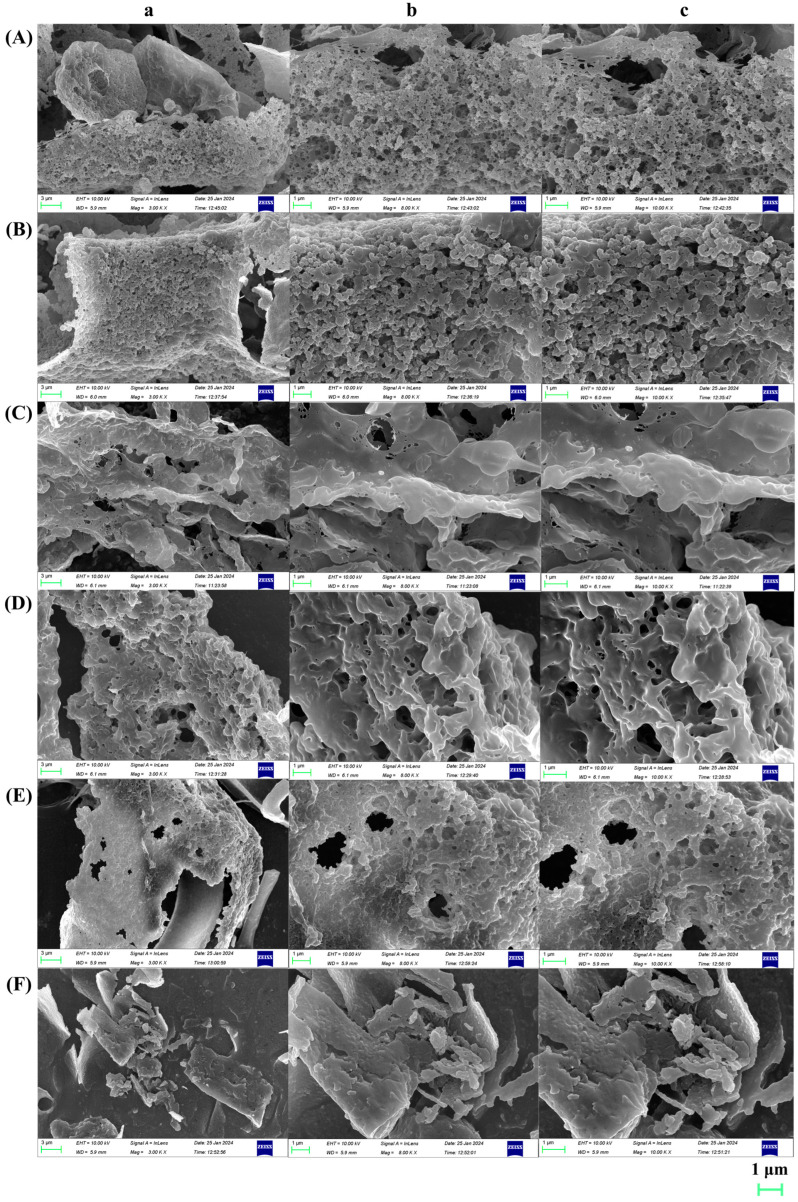
SEM photos of (**A**) SPS-L1, (**B**) SPS-L2, (**C**) SPS-R1, (**D**) SPS-R2, (**E**) SPS-S1, and (**F**) SPS-S2. a: 3000× magnification; b: 8000× magnification; c: 10,000× magnification.

The surface morphologies of the six SPSs are displayed in Figure 3, and they obviously varied with the parts and extraction methods. There were more granular spheres on the surfaces of SPS-L1 and SPS-L2. Although SPS-R2 featured a porous, loose structure, SPS-R1 had a surface that was comparatively smooth and compact. While the surface of SPS-S2 was smooth and fragmented, that of SPS-S1 was porous, small, and spherical with a tightly packed surface. The aforementioned findings demonstrated that the application of an ultrasonic wave resulted in the breakdown of the intramolecular hydrogen connection, a reduction in the rigidity of the dense polymer surface, and the fragmentation of the polysaccharide aggregation into pieces with a low value of *M_w_* [54].

#### 2.2.6. Congo Red Assay of the SPSs

**Figure 4:**
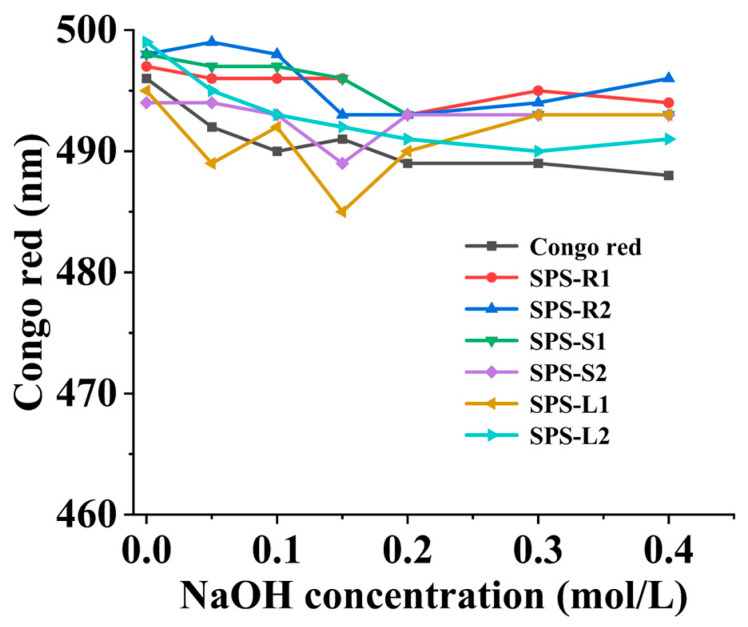
Three-helix conformation analysis of SPSs.

Congo red can be used to identify the three helical structures of a polysaccharide. When a polysaccharide forms a complex with Congo red in NaOH solution, the maximum absorption wavelength is redshifted [55]. Figure 4 shows the changes in the maximum absorption wavelength of the six kinds of SPSs and Congo red in NaOH solutions with different concentrations (0 to 0.4M). Compared to Congo red, the maximum absorption wavelengths of the six SPSs did not show a large redshift as the concentration of NaOH increased, indicating that they did not have a triple helix structure [22]. Studies have shown that the *M_w_*, composition of monosaccharides, and extraction techniques, which frequently impact the functional characteristics and biological activities of polysaccharides, are strongly correlated with their spiral structures [56].

#### 2.2.7. Thermal Analysis of the SPSs

**Figure 5:**
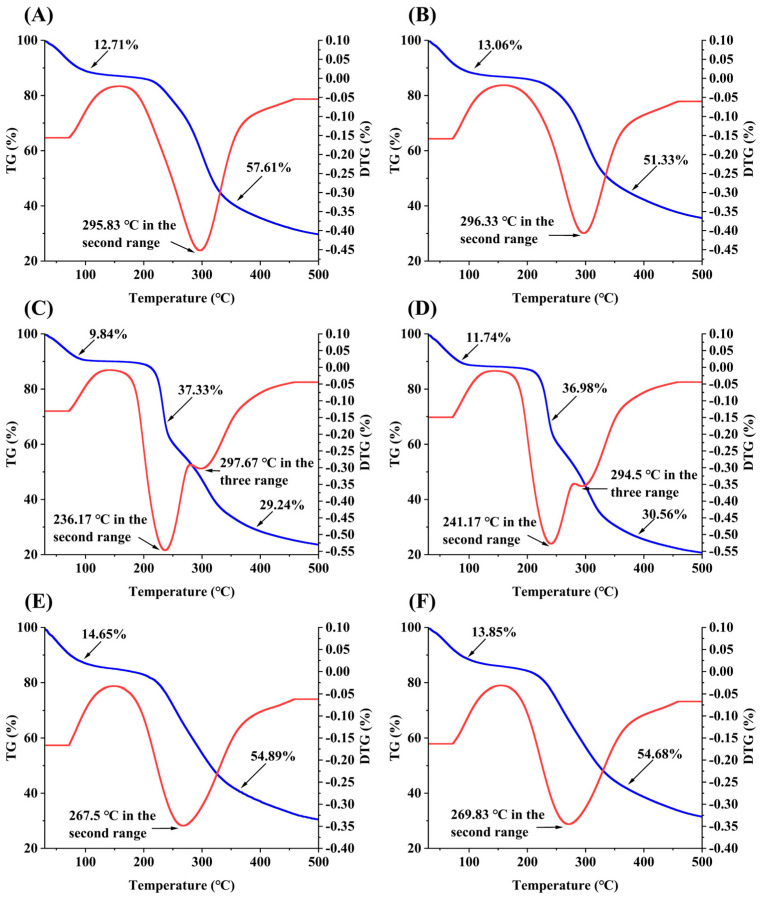
The TG-DTG curves of SPSs. (**A**) SPS-L1; (**B**) SPS-L2; (**C**) SPS-R1; (**D**) SPS-R2; (**E**) SPS-S1; and (**F**) SPS-S2. Blue line: TG; red line: DTG.

Figure 5 displays the results of thermogravimetric (TG) and derivative thermogravimetry (DTG) analyses of the SPSs. The TG-DTG curves of SPS-R1 and SPS-R2 obtained in three phases are depicted in Figure 5C,D. In the three phases, the temperatures were 30 °C to 140 °C, 140 °C to 280 °C, and 280 °C to 500 °C, and the weight loss rates were 9.84% and 11.74%, 37.33% and 36.98%, and 29.24% and 30.56%, respectively. The maximum weight loss temperatures in the second and third stages occurred at 236.17 °C and 241.17 °C and at 297.67 °C and 294.5 °C, respectively. The second stage had the highest weight loss of the three stages. In the first stage, weight was lost because the water in the polysaccharides evaporated; in the second stage, weight was lost because the polysaccharides depolymerized; in the third stage, weight was lost because organic matter was oxidatively decomposed. Similar TG-DTG curves were found for SPS-L1 and SPS-L2 and for SPS-S1 and SPS-S2 (Figure 5A,B,E,F); the mass loss in the first stage occurred between 30 °C and 150 °C, with loss rates of 12.71%, 13.76%, 14.65%, and 13.85%, respectively. Significant losses transpired in the second stage (150 °C to 500 °C), with loss rates of 57.61%, 51.33%, 54.89%, and 54.68%, respectively. SPS-L1 and SPS-S1 lost more in the second stage than SPS-L2 and SPS-S2 did. This showed that in comparison with the polysaccharides extracted using hot water, the polysaccharides extracted using ultrasound exhibited superior thermal stability [57]. The DTG curve showed that the maximum weight loss temperatures of SPS-L1, SPS-L2, SPS-S1, and SPS-S2 were 295.83 °C, 296.33 °C, 267.5 °C, and 269.83 °C, respectively. During the entire heating process (30 °C to 500 °C), the weight loss of SPS-R1 and SPS-R2 was higher than that of the other SPSs, which was due to the low *M_w_* of the polysaccharides from the roots, and the lower the *M_w_*, the lower the thermal stability [58].

### 2.3. In Vitro Antioxidant Activity of the SPSs

#### 2.3.1. DPPH Radical Scavenging Assay

**Figure 6:**
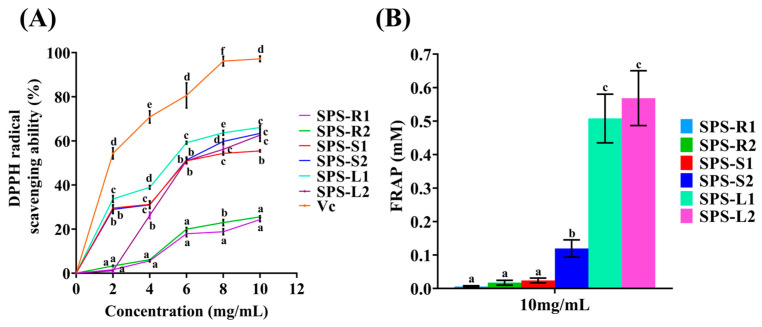
Antioxidant activities of SPSs. (**A**) DPPH radical scavenging activity; (**B**) FRAP assay. The above values are expressed as mean ± SD (*n* = 3). Different letters (a, b, c, d, e, and f) at the same concentration indicate a statistically significant difference (*p* < 0.05).

The impact of oxidative stress was mostly alleviated by scavenging free radicals [59]. As shown in Figure 6A, the six SPSs samples demonstrated the ability to clear DPPH free radicals in a dose-dependent manner. The DPPH free radical scavenging rates of SPS-L1, SPS-L2, SPS-R1, SPS-R2, SPS-S1, and SPS-S2 at a concentration of 10 mg/mL were 66.04%, 62.58%, 24.30%, 25.52%, 63.39%, and 55.44%, respectively. SPS-S1, SPS-S2, SPS-L1, and SPS-L2 demonstrated superior DPPH scavenging performance to that of SPS-R1 and SPS-R2.

#### 2.3.2. Ferric-Reducing Antioxidant Power (FRAP)

Determining the reducing power is a common way to test the antioxidant capabilities of natural products and is a crucial predictor of their antioxidant efficacy [60]. The main mechanism is the reduction of Fe^3+^ to Fe^2+^ upon interaction with an agent possessing antioxidant activity. In comparison with those of SPS-R1, SPS-R2, SPS-S1, and SPS-S2, the FRAP values of SPS-L1 and SPS-L2 at 10 mg/mL were substantially higher at 0.51 and 0.57 mM, respectively (Figure 6B). It is probable that the protein and pigment content of SPS-L1 and SPS-L2 was higher.

#### 2.3.3. Structure–Antioxidant Activity Relationship

The content of uronic acid, *M_w_*, monosaccharide composition, and sugar sequence linkage are closely related to the antioxidant activity of polysaccharides [61]. Li et al. reported that crude polysaccharides (CZSPs) isolated from *Zizyphus Jujuba* cv. *Jinsixiaozao* and purified components (ZSP4b, ZSP3C, ZSP2, and ZSP1b) might be used as electron or hydrogen donors to scavenge DPPH [62]. CFDP-T, which was isolated by Yan et al. from an industrial distillate of *Corbicula fluminea*, had a stronger scavenging ability for OH free radicals, which might have been due to the enhanced ability of CFDP-T to provide electrons or hydrogen atoms [49]. Studies have used bond dissociation enthalpy, ionization potential, proton dissociation enthalpy, proton affinity, and electron transfer enthalpy to explore three antioxidant mechanisms: hydrogen atom transfer, single-electron transfer-proton transfer, and sequential proton loss electron transfer. It was found that C-H bonds could effectively remove free radicals in the antioxidation process, and OH was more inclined to provide H atoms to free radicals [63,64]. SPS-L1, SPS-L2, SPS-S1, and SPS-S2 might have acted as stronger electrons or hydrogen atoms to clear DPPH. In our study, the *M_w_* values of the dominant components of SPS-L1, SPS-L2, SPS-S1, and SPS-S2 were higher than those of SPS-R1 and SPS-R2; polysaccharides with large values of *M_w_* provide a strong hydrogen supply capacity, thus showing excellent DPPH free radical scavenging ability [65]. The amounts of Rha and Gal, the sugar-binding pattern, and the polysaccharide structure were all strongly correlated with the antioxidant activity of polysaccharides [66]. In our study, the Rha and Gal contents of the polysaccharides from leaves and stems were higher than those of polysaccharides from roots.

Based on the experimental results for DPPH and FRAP, the polysaccharides from the leaves showed good antioxidant capacity, providing a foundation for utilizing non-medicinal parts of *Stemona tuberosa* Lour as antioxidants. The extract of *Stemona tuberosa* Lour can be used in the cosmetic industry, and *Stemona tuberosa* Lour itself is also a medicinal plant. Therefore, SPS-L1 and SPS-L2 may be used as potential natural sources of alternative additives in the cosmetic and pharmaceutical industries [67].

### 2.4. In Vitro Anti-Inflammatory Activity of the SPSs

**Figure 7:**
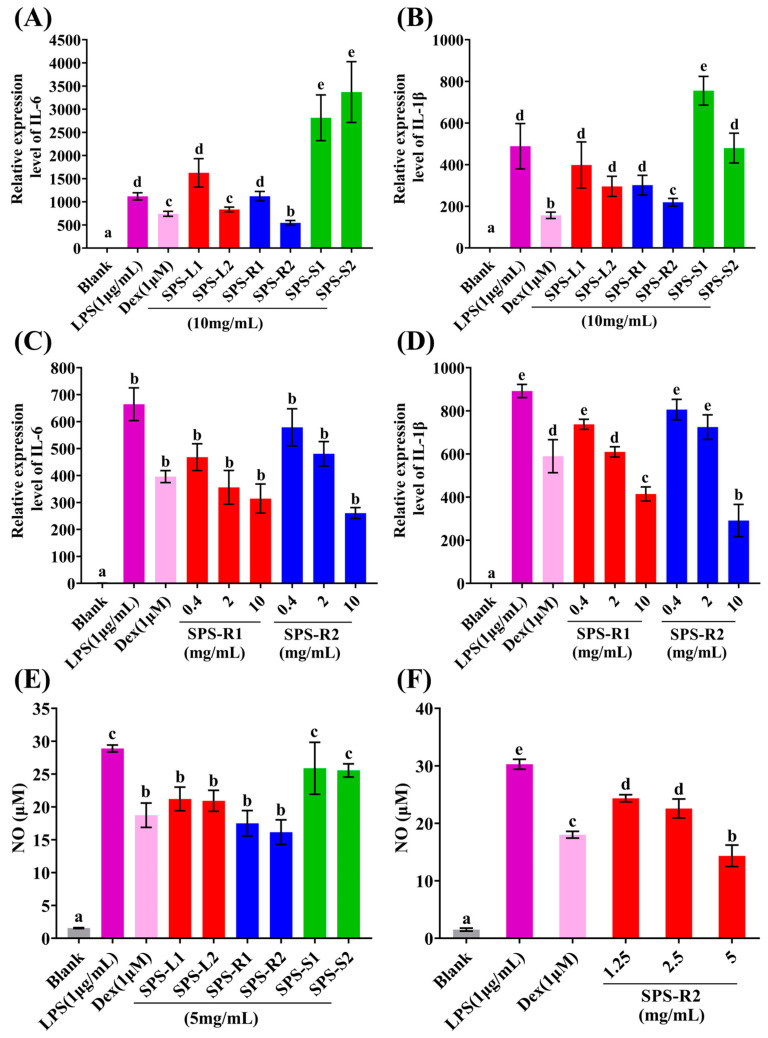
Anti-inflammatory activities of SPSs. The relative expression of IL-6 (**A**) and IL-1β (**B**) at 10 mg/mL of SPSs; the relative expression of IL-6 (**C**) and IL-1β (**D**) at 0.4–10 mg/mL of SPS-R1 and SPS-R2; the NO production at 5 mg/mL of SPSs (**E**); the NO production at 1.25–5 mg/mL of SPS-R2 (**F**). Bars with different letters are statistically different (*p* < 0.05). The above values are expressed as mean ± SD (*n* = 3).

Cytokines are important mediators in many physiological and pathological processes, such as inflammatory and immunological reactions [68]. When macrophage cells are activated by lipopolysaccharides (LPSs), they induce the expression of multiple inflammatory mediators and factors, such as NO, IL-6, IL-1β, and TNF-α [69]. Thus, the anti-inflammatory effect of the SPSs against RAW264.7 macrophages was assessed by detecting the NO production and the expression of IL-6 and IL-1β. SPS-R2 significantly downregulated IL-6 and IL-1β expression (*p* < 0.05) compared with SPS-R1, SPS-L1, SPS-L2, SPS-S1, and SPS-S2 (Figure 7A,B). In addition, gradient concentration (0.4–10 mg/mL) experiments showed that SPS-R1 and SPS-R2 decreased the expression of IL-6 and IL-1β in a dose-dependent manner (Figure 7C,D). At 10 mg/mL, SPS-R2 significantly (*p* < 0.05) downregulated IL-1β expression compared with SPS-R1 (Figure 7D). Compared with the other SPSs, SPS-R2 significantly (*p* < 0.05) downregulated NO production in a dose-dependent manner (Figure 7E,F). Notably, SPS-R2 had a higher total sugar content, which was associated with better anti-inflammatory activity [70,71]. Furthermore, the anti-inflammatory effects of the polysaccharides were significantly affected by their *M_w_* and monosaccharide composition [72]. SPS-R1, SPS-L1, SPS-L2, SPS-S1, and SPS-S2 had dominant components with higher values of *M_w_* than SPS-R2 did. This could have been because an increase in *M_w_* caused the solution to become more viscous, which, in turn, increased the mass transfer resistance of the polysaccharide and inhibited its binding to inflammatory cell receptors [73]. Glc was the primary component of two polysaccharides that were separated and refined from *Astragalus membranaceus* by Chen et al., and it demonstrated a strong anti-inflammatory effect [74]. The primary monosaccharide component of SPS-R1 and SPS-R2 in our investigation was Glc. A correlation was identified between the amount of Gal and anti-inflammatory properties in polysaccharides [75]. In addition, higher percentages of Man, Ara, and Rha also contributed to anti-inflammatory activity [76]. The aforementioned findings demonstrated the remarkable anti-inflammatory properties of SPS-R1 and SPS-R2, with the strongest anti-inflammatory properties coming from the ultrasonic extraction of SPS-R2. These findings provide some support for the exploitation of therapeutic anti-inflammatory medications.

### 2.5. In Vitro Effect of the SPSs on MUC5AC

**Figure 8:**
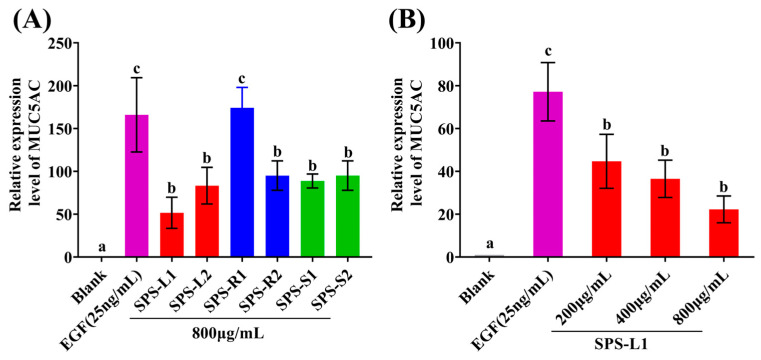
Effect of SPSs on hypersecretion model of NCI-H292 cells in vitro. The relative expression level of MUC5AC at 800 μg/mL of SPSs (**A**); the relative expression level of MUC5AC at 200–800 μg/mL of SPS-L1 (**B**). Bars with different letters are statistically different (*p* < 0.05). The above values are expressed as mean ± SD (*n* = 3).

The effects of the SPSs on the relative expression level of MUC5AC are shown in Figure 8. Figure 8A shows that SPS-L1, SPS-L2, SPS-R2, SPS-S1, and SPS-S2 significantly (*p* < 0.05) downregulated the expression of MUC5AC compared to the EGF group. Compared with the other SPSs, SPS-L1 showed a stronger ability to downregulate MUC5AC expression. Subsequently, a concentration gradient (200–800 μg/mL) experiment was performed on SPS-L1, and SPS-L1 significantly (*p* < 0.05) downregulated the expression of MUC5AC in a dose-dependent manner (Figure 8B). A previous study reported that extracts from *Ginkgo biloba*—kaempferol and quercetin—significantly downregulated MUC5AC expression [77]. SPS-L1 and SPS-L2 had a favorable ability to downregulate MUCAC expression, and MUCAC is closely related to the occurrence of many respiratory diseases (such as rhinitis and asthma) [78]. Therefore, in the future, SPS-L1 and SPS-L2 may play a role in the treatment of respiratory diseases.

## 3. Materials and Methods

### 3.1. Biological Materials and Chemicals

Fresh *Stemona tuberosa* Lour was provided by Guangdong Huakangyuan Medicinal Materials Resources Development Co., Ltd. (Qingyuan, China), dried at 60 °C, crushed into powder, and stored at −4 °C. DPPH and a Total Antioxidant Capacity Detection Kit (FRAP method) were purchased from Shanghai Enzyme-linked Biotechnology Co., Ltd. (Shanghai, China) and Shanghai Beyotime Biotechnology Co., Ltd. (Shanghai, China). LPSs (*Escherichia coli* O111:B4) were purchased from Sigma-Aldrich Chemical Co. (St Louis, MO, USA). EGF, cell culture medium 1640, fasting blood sugar (FBS), and qPCR reagents were purchased from Nanjing Vazyme Biotechnology Co., Ltd. (Nanjing, China). All other chemicals and solvents were of laboratory grade and were used directly.

### 3.2. Polysaccharide Extraction and Isolation

One gram of powder was subjected to Soxhlet extraction reflux with anhydrous ethanol for 1 day at 95 °C; the monosaccharides and fats were removed, and the powder was naturally dried after reflux, followed by extraction at 80 °C for 2 h three times at a solid-liquid ratio of 1:30 (g/mL). The supernatant was obtained through centrifugation at 4000 rpm for 10 min. It was concentrated to 30 mL; then, four times the volume of anhydrous ethanol was added, and it was kept at 4 °C overnight. The precipitation was obtained through centrifugation at 4000 rpm for 10 min and dissolved with distilled water, and the protein was removed by freezing and thawing repeatedly three times. The supernatant was collected via centrifugation and decolorized with HPD600 macroporous resin. SPS-L1, SPS-S1, and SPS-R1 were obtained by freeze drying the supernatant after 48 h of dialysis (MW: 3500 Da) in distilled water. SPS-L2, SPS-S2, and SPS-R2 were extracted in an 80 °C ultrasonic water bath (KQ-400KDE, Kun Shan Ultrasonic Instruments Co., Ltd., Suzhou, China) for 2 h three times; the other conditions and variables were consistent with those in the preparation of SPS-L1, SPS-S1, and SPS-R1.

### 3.3. Chemical Composition Analysis

The contents of total sugars, proteins, and uronic acid of the SPSs were determined with the phenol-sulfuric acid method [79], Bradford method [80], and sulfate-carbazole method [81], respectively. Glucose, bovine serum albumin (BSA), and galacturonic acid were used as standards, respectively. The yield of the SPSs (EY, %) was expressed as

EY (%) = M_1_/M_2_ × 100%

where M_1_ is the mass of the lyophilized SPSs and M_2_ is the quality of the raw powder.

### 3.4. Determination of the Molecular Weight and Analysis of Monosaccharide Composition

The SPSs were properly weighed to 5 mg and dissolved in the mobile phase (0.05 mol/L NaCl solution) to obtain a 5 mg/mL solution. High-performance gel permeation chromatography (HPGPC) was used to evaluate the solution. A column temperature of 40 °C and a flow rate of 0.8 mL/min were used. A BRT105-103-101 tandem gel column (8 × 300 mm) and an RID-20A difference detector (Shimadzu, kyoto, Japan) were used in the HPGPC system [82].

The precision of the experiment involved weighing 5 mg of the SPSs, followed by the addition of 2 mL of 2 mol/L TFA. The mixture was then hydrolyzed at a temperature of 120 °C for a duration of 2 h. Subsequently, the sample was dried using nitrogen gas and vortexed with deionized water to ensure thorough mixing. The measurement of high-performance ion exchange chromatography (HPIC) was conducted using a Dionex Carbopac™ PA20 column (3 × 150 mm, Thermo Fisher Scientific, Waltham, MA, USA) and an electrochemical detector [83].

### 3.5. UV Spectroscopy Analysis and FT-IR Spectroscopic Analyses

An ultraviolet-visible (UV-VIS) spectrophotometer (NP80 Touch, Implen, Munich, Germany) was used to scan the SPSs at a concentration of 0.1 mg/mL in the range of 200–500 nm.

The Fourier-transform infrared (FT-IR) spectra of the SPSs over 450–4500 cm^−1^ were measured using a TENSOR 27 FT-IR spectrometer (Bruker Corporation, Saarbrucken, Germany).

### 3.6. Scanning Electron Microscopy (SEM)

The surface morphology of the SPSs was observed with a scanning electron microscope (Sigma-300, Carl Zeiss AG, Oberkochen, Germany).

### 3.7. Congo Red Assay of the SPSs

The final concentrations of NaOH were 0, 0.05, 0.1, 0.15, 0.2, 0.3, and 0.4 M, respectively, when the SPSs with a concentration of 1 mg/mL were mixed with 80 μM Congo red solution, and 1 mol/L NaOH solution was added [84]. After being left to stand for 5 min, the maximum absorption wavelength of the solution was determined with a UV-VIS (NP80 Touch, Implen, Munich, Germany) spectrophotometer in the range of 400–600 nm.

### 3.8. Thermal Analysis of the SPSs

The thermal properties of the SPSs were studied using a thermal analyzer (Mettler TGA/DSC3+, Zurich, Switzerland) produced by Mettler Toledo based on TG-DTG. In short, 10 mg of the SPSs was added to Al_2_O_3_, and empty aluminum was used as a raw material. The experiment was carried out at a heating rate of 10 °C/min in a N_2_ environment with a temperature of 30 to 500 °C [85].

### 3.9. In Vitro Antioxidant Activity

#### 3.9.1. DPPH Radical Scavenging Assay

According to the method used for the kit provided by Shanghai Enzyme-linked Biotechnology Co., Ltd., 0.10 mL of SPS (2–10 mg/mL) solution was mixed with a working liquid (0.10 mL) and then mixed well. The absorbance was measured at 515 nm using a microplate reader (Tecan M2001,Tecan, Mannedorf, Switzerland) after being left to stand at room temperature for 20 min away from light [86]. Vitamin C (Vc) was used as a positive control. The calculation formula was as follows:

DPPH radical scavenging rate (%) = ((A_1_ − A_2_)/A_1_) × 100%

where A_1_ represents the absorbance value of the blank control in the working fluid, and A_2_ represents the absorbance value of the sample in the working fluid.

#### 3.9.2. Ferric-Reducing Antioxidant Power

According to the protocol of the Total Antioxidant Capacity Detection Kit from Beyotime Biotechnology (FRAP method) [87], 5 μL of the SPSs (10 mg/mL) was incubated in 180 μL of FRAP working solution at 37 °C for 5 min, and the absorbance was detected at 593 nm. The antioxidant capacity of the SPSs was calculated according to the FeSO_4_ standard curve.

### 3.10. In Vitro Anti-Inflammatory Activity

#### 3.10.1. Effects on the Secretion of IL-6 and IL-1β from RAW264.7 Macrophages

RAW264.7 macrophages were seeded in 12-well plates cultured at 37 °C with 5% CO_2_ until 80% growth. The groups were a normal control group (without LPS stimulation), an LPS stimulation group (1 μg/mL), and an administration group (10 mg/mL SPSs + 1 μg/mL LPS). A dexamethasone group (DEX, 1 μM) was used as a positive control. After induction with 1 μg/mL LPS for 4 h, RNA was extracted, and cDNA was prepared using real-time fluorescence quantitative PCR according to the method from Vazyme [88]. The primers were synthesized by Sangon Biotech (Shanghai) Co., Ltd. (Shanghai, China), and the sequences are shown in the Supplementary Material (Table S1).

#### 3.10.2. Effects of NO Production on RAW264.7 Macrophages

After an overnight culture in a 96-well plate (2 × 10^4^ cells/well), the cells were pre-treated with the SPSs or DEX for 1 h. Then, 1 μg/mL LPS was added for an additional 12 h. The supernatant was collected from each well. The NO production was measured using a NO assay kit (Beyotime Biotechnology) according to the manufacturer’s instructions, and the absorbance was measured at 540 nm using a microplate reader [89].

### 3.11. Effects of the SPSs on High Secretion in NCI-H292 Cells

NCI-H292 cells were cultured in 1640 medium (containing 10% FBS) and 5% CO_2_ and placed in a 37 °C incubator. The medium was changed to 1640 medium without FBS when the cells reached 80% growth to starve them. The SPSs were added at concentrations of 200 μg/mL, 400 μg/mL, and 800 μg/mL SPSs. After 1 h of cultivation, 25 ng/mL EGF was added. After 24 h, RNA was extracted using the TRIZOL method. The expression of the MUC5AC gene was measured using qPCR [90]. The primers were synthesized by Sangon Biotech (Shanghai) Co., Ltd., and the sequences are shown in the Supplementary Material (Table S1).

### 3.12. Statistical Analysis

All data are shown as the mean ± standard deviation (SD). SPSS 27.0 software (SPSS, Chicago, IL, USA) was used for statistical analysis. Tukey’s multiple-comparison test and one-way analysis of variance (ANOVA) were used to compare the mean values between groups. Values with *p* < 0.05 were considered statistically significant.

## 4. Conclusions

In conclusion, SPS-L1, SPS-L2, SPS-R1, SPS-R2, SPS-S1, and SPS-S2 were extracted from the leaves, roots, and stems of *Stemona tuberosa* Lour using hot water and ultrasound-assisted methods. The findings demonstrated that there were notable differences in the physicochemical characteristics, *M_w_*, monosaccharide composition, surface shape, thermal stability, antioxidant activity, anti-inflammatory activity, and MUC5AC expression across the various extraction methods and sections of the plant from which the SPSs were obtained. SPS-R1 and SPS-R2, which were obtained from the medicinal part of the roots, had an excellent inhibitory effect on inflammation, and they can be further used in the development of clinical anti-inflammatory drugs. SPS-L1 and SPS-L2 from the leaves may be used as potential natural antioxidants and may also be further studied in the treatment of diseases. In this study, polysaccharides were extracted from different parts of *Stemona tuberosa* Lour with different extraction methods, and the structural characterization and activity of polysaccharides were studied, which can offer valuable insights for future research and the development of polysaccharides. However, due to the complex and diverse nature of polysaccharides, further analysis of their purification, structure, and in vivo anti-inflammatory activity is needed for understanding the relationship between activity and polysaccharide structure.

## Figures and Tables

**Table 1:** The extraction yield and chemical properties of six SPSs were obtained by hot water extraction and ultrasonic-assisted extraction.

SPSs	Yield (%)	Total Sugar (%)	Protein (%)	Uronic Acid (%)
SPS-L1	10.49 ± 0.61 ^a^	34.06 ± 1.99 ^d^	4.83 ± 0.26 ^d^	17.91 ± 0.95 ^c^
SPS-L2	8.75 ± 0.27 ^b^	23.30 ± 1.28 ^e^	9.24 ± 0.41 ^b^	14.37 ± 0.91 ^d^
SPS-R1	11.75 ± 1.10 ^a^	89.98 ± 2.88 ^a^	1.07 ± 0.18 ^e^	6.42 ± 0.44 ^e^
SPS-R2	11.46 ± 1.04 ^a^	80.50 ± 1.80 ^b^	0.94 ± 0.11 ^e^	13.09 ± 1.90 ^d^
SPS-S1	7.18 ± 0.25 ^c^	42.23 ± 1.11 ^c^	8.37 ± 0.17 ^c^	50.39 ± 1.39 ^a^
SPS-S2	4.96 ± 0.67 ^d^	41.18 ± 0.66 ^c^	10.10 ± 0.17 ^a^	42.04 ± 1.89 ^b^

The SPS-L1, SPS-L2, SPS-R1, SPS-R2, SPS-S1, and SPS-S2 obtained from the leaves, roots, and stems of the *Stemona tuberosa* Lour by hot water extraction and ultrasonic-assisted extraction. Data are represented as mean value ± SD (*n* = 3); values of different letters in the same column indicate significant differences (*p* < 0.05).

**Table 2:** *M_w_* distribution of SPSs.

Sample	Peak	Retention Time (min)	*M_w_* (Da) ^a^	*M_n_* (Da) ^b^	*M_w_*/*M_n_* ^c^	Mass Fraction (%)
SPS-L1	1	29.64	88,093	86,088	1.02	36.98
	2	33.56	11,098	10,669	1.04	63.02
SPS-L2	1	29.65	87,478	85,487	1.02	17.75
	2	33.60	10,834	10,414	1.04	82.25
SPS-R1	1	30.91	161,027	114,627	1.41	12.20
	2	35.11	5575	3261	1.71	87.80
SPS-R2	1	28.51	161,943	158,257	1.02	11.66
	2	35.84	3120	3049	1.02	88.34
SPS-S1	1	27.72	248,136	242,488	1.02	11.09
	2	31.80	27437	26812	1.02	37.65
	3	32.49	18,918	18,487	1.02	17.55
	4	33.06	13,908	13,591	1.02	24.19
	5	35.48	3792	3706	1.02	9.52
SPS-S2	1	28.67	148,327	144,951	1.02	17.18
	2	31.40	34,035	33,261	1.02	24.50
	3	33.56	10,669	10,426	1.02	58.32

^a^ Weight average molecular weight, ^b^ number average molecular weight, ^c^ polydispersity.

**Table 3:** Monosaccharide composition of SPSs (%).

Sample	SPS-L1	SPS-L2	SPS-R1	SPS-R2	SPS-S1	SPS-S2
Fuc	nd	nd	0.13	nd	nd	nd
Rha	2.4	4.05	1.80	1.01	4.06	3.80
Ara	9.25	10.43	4.03	5.05	5.09	5.29
Gal	27.89	29.48	17.71	12.22	20.61	22.30
Glc	8.79	11.86	69.86	55.71	3.09	4.01
Xyl	2.02	2.00	nd	nd	2.50	2.94
Man	25.39	25.03	nd	nd	13.35	14.78
Rib	nd	nd	nd	nd	nd	nd
GalA	23.14	15.05	2.32	26.02	48.82	44.62
GlcA	0.90	1.59	1.26	nd	2.21	1.95

nd: not detected. Fuc: fucose; Rha: rhamnose; Ara: arabinose; Gal: galactose; Glc: glucose; Xyl: xylose; Man: mannose; Rib: ribose; GalA: galacturonic acid; GlcA: glucuronic acid.

## Data Availability

Data are contained within the article.

## Supplementary Materials

The following supporting information can be downloaded at: https://www.mdpi.com/article/10.3390/molecules29061347/s1, Table S1: Primers used in this study.

## Abbreviations

MUC5AC	Mucin 5AC
SPSs	Polysaccharides extracted from *Stemona tuberosa* Lour
SPS-L1, SPS-R1, and SPS-S1	Polysaccharides extracted from *Stemona tuberosa* Lour’s leaves, roots, and stems with hot water
SPS-L2, SPS-R2, and SPS-S2	Polysaccharides extracted from *Stemona tuberosa* Lour’s leaves, roots, and stems with ultrasonic-assisted method
*M_w_*	Weight average molecular weight
NO	Nitric oxide
IL-6	Interleukin-6
IL-1β	Interleukin-1β
TNF-α	Tumor necrosis factor-α
ROS	Reactive oxygen species
DPPH	2,2-diphenyl-1-picrylhydrazyl
ABTS	2, 2’-azino-bis(3-ethylbenzothiazoline-6-sulfonic acid)
LPS	Lipopolysaccharide
EGF	Epidermal growth factor
Fuc	Fucose
Rha	Rhamnose
Ara	Arabinose
Gal	Galactose
Glc	Glucose
Xyl	Xylose
Man	Mannose
Rib	Ribose
GalA	Galacturonic acid
GlcA	Glucuronic acid
TG	Thermogravimetric
DTG	Derivative thermogravimetry
FRAP	Ferric-Reducing Antioxidant Power
