# Supplementary material for: Study of the Structure and Bioactivity of Polysaccharides from Different Parts of Stemona tuberosa Lour
Source: Molecules. 2024 Mar 18;29(6):1347. doi: 10.3390/molecules29061347 (PMC10975339; doi:10.3390/molecules29061347)
Supplement: Supplementary file 1 [file molecules-29-01347-s001.zip › molecules-2900841-supplementary.pdf]

Table S1 Primers used in this study.

Target gene	Forward sequence(5'→3')	Reverse sequence(5'→3')
IL-1β	TCGTGCTGTCGGACCCATAT	GTCGTTGCTTGGTTCTCCTTGT
IL-6	TACTCGGCAAACCTAGTGCG	GTGTCCCAACATTCATATTGTCAGT
MUC5AC	CTGTGAAGGTGGCTGACCAAGA	AAGGTGTAGTAGGTGCCGTCGAA
β-Actin	GGCTGTATTCCCCTCCATCG	CCAGTTGGTAACAATGCCATGT
